# Association of Vitamin D Deficiency With Glycemic Control in Type 2 Diabetes Mellitus

**DOI:** 10.7759/cureus.99160

**Published:** 2025-12-13

**Authors:** Anwar Ul Haq, Muhammad Bilal Khattak

**Affiliations:** 1 Internal Medicine, Hayatabad Medical Complex Peshawar, Peshawar, PAK; 2 Medicine, Khyber Girls Medical College, Peshawar, PAK

**Keywords:** glycated hemoglobin a, insulin resistance, outcome, type 2 diabetes mellitus, vitamin d deficiency

## Abstract

Background

Insulin secretion and glucose metabolism are influenced by vitamin D. Deficiency can lead to a decrease in insulin sensitivity and a deterioration in glycemic control.

Objectives

This study’s objective is to examine the association between serum vitamin D and glycemic control (HbA1c) among patients with type 2 diabetes mellitus (T2DM).

Methods

We studied 120 patients with T2DM. The measurement of vitamin D (25-hydroxyvitamin D (25(OH)D)) and HbA1c used established methods. Deficiency was classified as having a level of vitamin D < 20 ng/mL.

Results

Mean age was 52.6 ± 9.4 years; 70/120 (58.3%) were male. Vitamin D deficiency was present in 82/120 (68.3%). Mean HbA1c was higher in deficient vs sufficient patients (8.4 ± 1.2% vs 7.6 ± 1.0%, t = 3.82, p = 0.002). Fasting plasma glucose and Homeostatic Model Assessment for Insulin Resistance (HOMA-IR) were also higher in the deficient group (p ≤ 0.01). Serum vitamin D correlated negatively with HbA1c (r = −0.34, p = 0.001).

Conclusion

Vitamin D deficiency was common and associated with poorer glycemic control. Screening and treatment of deficiency may aid metabolic outcomes in T2DM.

## Introduction

Type 2 diabetes mellitus (T2DM) is a chronic metabolic disorder characterized by insulin resistance and persistent hyperglycemia, leading over time to microvascular and macrovascular complications. Its global prevalence is increasing, with a particularly rapid rise in low- and middle-income countries, including those in South Asia. Pakistan has reported a high and growing burden of T2DM, placing considerable strain on healthcare systems and underscoring the importance of identifying potentially modifiable factors associated with glycemic control [1-3].

Beyond its classical role in calcium-phosphate homeostasis and bone metabolism, vitamin D is now recognized as a pleiotropic hormone involved in immune regulation, inflammation, and glucose metabolism. Vitamin D receptors are expressed in pancreatic β-cells and insulin-responsive tissues such as skeletal muscle and adipose tissue, suggesting biological pathways through which vitamin D status may influence insulin secretion and sensitivity [4-6].

Observational and interventional studies have reported associations between lower serum 25-hydroxyvitamin D (25(OH)D) concentrations and higher fasting glucose, HbA1c, and indices of insulin resistance, although the strength and consistency of these relationships vary across populations [7-10].

In South Asia, including Pakistan, vitamin D deficiency is highly prevalent and has been attributed to limited sun exposure, cultural clothing practices, darker skin pigmentation, and suboptimal dietary intake [11-13]. Given the simultaneous high burden of T2DM and vitamin D deficiency in this region, understanding their relationship has important clinical and public-health implications. However, local data from Pakistani populations remain limited, and the extent to which vitamin D status is associated with glycemic control and insulin resistance in this setting is not fully established. Therefore, this study aimed to determine the association between serum 25(OH)D levels and glycemic control, assessed by HbA1c, in patients with T2DM, and to assess whether vitamin D deficiency correlates with elevated HbA1c and increased insulin resistance as estimated by the Homeostatic Model Assessment for Insulin Resistance (HOMA-IR).

Research objective

The objective of this study was to determine the association between serum 25(OH)D levels and glycemic control, assessed through HbA1c, in adults with T2DM. A secondary objective was to examine whether vitamin D deficiency is correlated with increased insulin resistance as estimated by the homeostatic model assessment of insulin resistance (HOMA-IR).

## Materials and methods

Study design and setting

This cross-sectional study was conducted in the Department of Endocrinology, Hayatabad Medical Complex (HMC), Peshawar, Pakistan, from January to June 2024.

Participants and sampling

Adults aged 30-70 years with a known diagnosis of T2DM for at least one year were eligible. T2DM was diagnosed using American Diabetes Association (ADA) criteria (fasting plasma glucose ≥126 mg/dL, two-hour OGTT plasma glucose ≥200 mg/dL, HbA1c ≥6.5%, or current use of glucose-lowering therapy in a compatible clinical context).

Consecutive non-probability sampling was used; all eligible and consenting patients attending the outpatient endocrinology clinic during the study period were enrolled.

Exclusion criteria: type 1 diabetes, gestational diabetes, chronic liver disease, chronic kidney disease (eGFR <60 mL/minute/1.73 m²), known malignancy, pregnancy, disorders of calcium or vitamin D metabolism, vitamin D supplementation within the last three months, long-term corticosteroid therapy, or incomplete laboratory data for HOMA-IR.

Data collection and clinical measurements

Demographic and clinical data (age, sex, diabetes duration, antidiabetic medications, and comorbidities) were recorded using a structured proforma (Appendices).

Height and weight were measured using standardized equipment; BMI was calculated as kg/m².

Blood pressure was measured after five minutes of seated rest using an automated sphygmomanometer with an appropriately sized cuff. Two readings were obtained one to two minutes apart, and the average was recorded.

Laboratory measurements

Venous blood samples were collected in the morning after an eight- to 12-hour overnight fast.

Serum 25(OH)D was measured using a quantitative ELISA kit (DRG Instruments GmbH, Marburg, Germany; catalog EIA-5396).

HbA1c was measured using high-performance liquid chromatography (HPLC) (Tosoh G8 Analyzer, Tosoh Bioscience, Tokyo, Japan; IFCC-standardized).

Fasting plasma glucose, postprandial glucose, and lipid profile were analyzed using an automated chemistry analyzer (Cobas c 311, Roche Diagnostics, Mannheim, Germany).

Fasting insulin was measured using a chemiluminescent immunoassay (Architect i2000SR, Abbott Laboratories, Abbott Park, IL).

All analyses were conducted in the same hospital laboratory using routine internal quality control and external quality assurance procedures.

Estimation of insulin resistance

Insulin resistance was calculated using the homeostatic model assessment (HOMA-IR) formula:

HOMA-IR = (fasting insulin (µU/mL) × fasting glucose (mg/dL)) / 405

As all glucose values were recorded in mg/dL, this formula was applied consistently.

Sample size calculation

Sample size estimation for correlation analysis between serum 25(OH)D and HbA1c assumed a correlation coefficient of |r| = 0.30, α = 0.05, and power = 80%. The minimum sample required was 84 (Fisher’s z-transformation). To allow for missing data and subgroup analyses, the sample size was increased to 120.

Statistical analysis

Statistical analyses were performed using IBM SPSS Statistics version 24.0 (IBM Corp., Armonk, NY). Continuous variables were summarized as mean ± SD or median (IQR), and categorical variables as frequencies and percentages.

Normality was assessed using the Shapiro-Wilk test and visual inspection. Group comparisons used independent-samples t-tests, Welch’s t-tests, or Mann-Whitney U-tests as appropriate. Correlations between serum 25(OH)D and metabolic variables were assessed using Pearson’s or Spearman’s correlation coefficients. HbA1c categories (<7%, 7-8%, >8%) were compared using the chi-square test or Fisher’s exact test where applicable. An exploratory multivariable regression was considered to adjust for age, sex, BMI, and diabetes duration.

A p-value < 0.05 was considered statistically significant.

Ethical considerations

The study followed the Declaration of Helsinki. Ethical approval was obtained from the Institutional Review Board of HMC (Reference No. 1180/DOS/MTI/HMC). Written informed consent was obtained from all participants, including consent for open-access publication of anonymized data.

## Results

A total of 120 patients with T2DM were included in the analysis. The mean age was 52.6 ± 9.4 years, and 70 (58.3%) were male. Vitamin D deficiency (25(OH)D <20 ng/mL) was present in 82 (68.3%) participants. Baseline demographic and clinical characteristics by vitamin D status are shown in Table 1. Apart from BMI, which was higher in the vitamin D-deficient group, age, sex distribution, duration of diabetes, and blood pressure were broadly similar between groups.

**Table 1:** Baseline Demographic and Clinical Characteristics (n = 120) Continuous variables are presented as mean ± SD and were compared between groups using independent-samples t-tests (or Welch’s t-test where appropriate). Gender was compared using the chi-square test. BMI, body mass index; BP, blood pressure

Variable	Total (n = 120)	Vitamin D Deficient (n = 82)	Vitamin D Sufficient (n = 38)	p-value
Age (years), mean ± SD	52.6 ± 9.4	53.2 ± 8.9	51.4 ± 9.8	0.42
Gender (male/female), n	70 / 50	48 / 34	22 / 16	0.81
Duration of diabetes (years), mean ± SD	8.6 ± 4.5	8.8 ± 4.2	8.1 ± 4.9	0.56
BMI (kg/m²), mean ± SD	27.7 ± 3.2	28.4 ± 3.2	26.1 ± 2.8	0.03
Systolic BP (mmHg), mean ± SD	134.5 ± 14.3	136.1 ± 13.6	131.2 ± 15.1	0.11
Diastolic BP (mmHg), mean ± SD	84.2 ± 8.1	85.1 ± 7.8	82.6 ± 8.5	0.19

Biochemical parameters are presented in Table 2. Compared with vitamin D-sufficient participants, those with vitamin D deficiency had higher mean HbA1c, fasting plasma glucose, postprandial glucose, and HOMA-IR, indicating poorer glycemic control and higher estimated insulin resistance. Lipid profile parameters (total cholesterol, triglycerides, HDL-cholesterol) did not differ significantly between groups.

**Table 2:** Biochemical Parameters in Study Participants Values are presented as mean ± SD. Group comparisons were performed using independent-samples t-tests (or Welch’s t-test as appropriate). HDL-C, high-density lipoprotein cholesterol; HOMA-IR, homeostatic model assessment of insulin resistance

Parameter	Vitamin D Deficient (n = 82)	Vitamin D Sufficient (n = 38)	p-value
Serum 25(OH)D (ng/mL), mean ± SD	18.2 ± 7.6	29.5 ± 8.2	<0.001
HbA1c (%), mean ± SD	8.4 ± 1.2	7.6 ± 1.0	0.002
Fasting plasma glucose (mg/dL), mean ± SD	162.4 ± 28.5	142.6 ± 25.3	0.004
Postprandial glucose (mg/dL), mean ± SD	232.8 ± 45.6	205.2 ± 39.7	0.01
HOMA-IR, mean ± SD	3.7 ± 1.3	2.9 ± 1.0	0.01
Total cholesterol (mg/dL), mean ± SD	193.4 ± 32.8	184.7 ± 28.2	0.18
Triglycerides (mg/dL), mean ± SD	156.2 ± 42.1	142.3 ± 37.9	0.21
HDL-C (mg/dL), mean ± SD	41.6 ± 7.4	44.9 ± 8.2	0.09

Correlation analyses between serum 25(OH)D and metabolic variables are summarized in Table 3. Serum 25(OH)D showed weak inverse correlations with HbA1c, fasting plasma glucose, HOMA-IR, and BMI, whereas no clear association was observed with duration of diabetes. Although statistically significant, the magnitude of the correlations was modest, indicating that vitamin D status explained only a small proportion of the variability in these parameters.

**Table 3:** Correlation Between Serum 25(OH)D and Glycemic/Anthropometric Variables Variable Pearson’s correlation coefficients are presented; for variables that deviated substantially from normality, results were confirmed with Spearman’s rank correlation (not shown).

Variable	Correlation Coefficient (r)	p-value
HbA1c (%)	-0.34	0.001
Fasting plasma glucose (mg/dL)	-0.29	0.004
HOMA-IR	-0.25	0.01
BMI (kg/m²)	-0.21	0.03
Duration of diabetes (years)	-0.08	0.32

The distribution of glycemic control categories according to vitamin D status is shown in Table 4. Poor glycemic control (HbA1c >8%) was more frequent among vitamin D-deficient participants, whereas a higher proportion of vitamin D-sufficient participants fell into the good control category (HbA1c <7%). Group differences in HbA1c categories were statistically significant.

**Table 4:** Glycemic Control Categories by Vitamin D Status Comparison: χ² = 10.53, df = 2, p = 0.005 (chi-square test). HbA1c, glycated hemoglobin

HbA1c Category	Vitamin D Deficient (n = 82)	Vitamin D Sufficient (n = 38)
Good (<7%)	10 (12.2%)	12 (31.6%)
Fair (7-8%)	22 (26.8%)	14 (36.8%)
Poor (>8%)	50 (61.0%)	12 (31.6%)

## Discussion

In this cross-sectional investigation involving adults diagnosed with T2DM who were receiving care at a tertiary medical facility in Pakistan, a notable prevalence of vitamin D deficiency was identified, which correlated with indicators of suboptimal metabolic regulation. Participants exhibiting insufficient 25-hydroxyvitamin D (25(OH)D) levels demonstrated elevated HbA1c, fasting, and postprandial plasma glucose levels, as well as higher Homeostasis Model Assessment of Insulin Resistance (HOMA-IR) scores in comparison to individuals with adequate vitamin D status; furthermore, serum 25(OH)D concentrations displayed weak inverse correlations with glycemic measures and indicators of insulin resistance. Although the observed effect sizes were relatively modest, these outcomes align with prior observational research documenting an inverse association between vitamin D levels and glycemic control in populations afflicted with T2DM [7-10,14-16].

Beyond mere epidemiological correlations, substantial experimental and clinical evidence substantiates a biologically plausible connection between vitamin D3 and insulin resistance. The biologically active form, 1,25-dihydroxyvitamin D3, interacts with the intracellular vitamin D receptor (VDR), which is expressed in pancreatic β-cells, hepatocytes, skeletal muscle, and adipose tissue. The binding of the VDR to its ligand facilitates its heterodimerization with the retinoid X receptor (RXR), subsequently regulating the transcription of genes implicated in insulin production, insulin receptor signaling, and glucose transport. Preclinical investigations have revealed that the activation of VDR enhances insulin sensitivity through the modulation of insulin receptor substrate (IRS)-phosphoinositide 3-kinase (PI3K)-Akt signaling pathways within skeletal muscle and adipose tissue [17,18], whereas VDR deficiency has been shown to hinder first-phase insulin secretion and exacerbate insulin resistance. In mechanistic studies involving human subjects, repletion of vitamin D has been correlated with improvements in β-cell functionality and modest increases in insulin sensitivity, particularly among patients exhibiting profound deficiency [19].

Vitamin D3 additionally acts as an epigenetic modulator that influences insulin signaling pathways at the transcriptional level. The VDR-RXR complex directly binds to vitamin D response elements (VDREs) located within the promoter regions of genes that regulate IRSs, glucose transport, calcium channels, and inflammatory mediators. Epigenomic investigations indicate that vitamin D status modulates patterns of DNA methylation and histone acetylation at critical metabolic genes, including IRS1, IRS2, and components of the PI3K-Akt signaling cascade [20-21]. Furthermore, calcium homeostasis, which is essential for insulin exocytosis and downstream signaling, is impacted by vitamin D-mediated regulation of calcium channels and calcium-binding proteins [22-23]. Concurrently, vitamin D demonstrates antioxidant properties by upregulating endogenous antioxidant enzymes such as superoxide dismutase and glutathione peroxidase, while concurrently inhibiting pro-oxidative and pro-inflammatory pathways that contribute to β-cell dysfunction and insulin resistance [24-25]. Collectively, these preclinical and human findings suggest a convergence of multiple mechanisms - genomic, epigenetic, inflammatory, and oxidative - that may elucidate the association between vitamin D deficiency and impaired glycemic regulation.

The elevated prevalence of vitamin D deficiency documented within this cohort is consistent with existing literature from South Asian populations, including Pakistan, where factors such as insufficient sunlight exposure, cultural apparel practices, darker skin pigmentation, and inadequate dietary intake commonly contribute to this phenomenon [11-13]. Additionally, we observed that body mass index (BMI) was significantly elevated among participants with vitamin D deficiency, corroborating evidence that excess adiposity predisposes individuals to lower circulating vitamin D levels due to volumetric dilution or sequestration within adipose tissue. This observation raises the potential for BMI, lifestyle behaviors, and other uncontrolled variables to confound the relationship between vitamin D status and glycemic control. Given that our analyses were not adjusted, these associations warrant cautious interpretation and cannot definitively isolate the independent metabolic influence of vitamin D.

Importantly, although significant, the correlations between 25(OH)D and metabolic measures in this study were weak, indicating that vitamin D status is likely one of several factors influencing glycemic control and insulin resistance in individuals with T2DM. The cross-sectional design further limits causal inference. Patients with longstanding or poorly controlled diabetes, who often have reduced outdoor activity, higher adiposity, and altered dietary patterns, may be more likely to exhibit vitamin D deficiency, raising the possibility of reverse causation and residual confounding.

Overall, these findings contribute region-specific evidence from a Pakistani tertiary care setting to the broader literature on vitamin D and metabolic outcomes in T2DM. Future longitudinal and interventional studies, particularly randomized controlled trials that incorporate mechanistic biomarkers and epigenetic profiling, are needed to determine whether correction of vitamin D deficiency leads to meaningful improvements in glycemic regulation and to identify subgroups that may derive the greatest benefit from targeted vitamin D interventions.

Limitations 

This study has several important limitations. First, the cross-sectional design prevents the determination of temporal or causal relationships between vitamin D status and glycemic control; the observed associations may be influenced by reverse causation. Second, the sample was drawn from a single tertiary care center using consecutive sampling, which may limit generalizability to other settings and to patients with milder or untreated disease. Third, although we excluded major comorbid conditions and collected basic clinical information, we did not systematically account for potential confounders such as detailed antidiabetic medication regimens, physical activity, dietary intake, sunlight exposure, and seasonality, or fully adjust for BMI in multivariable analyses. These factors could plausibly influence both vitamin D status and glycemic control. Fourth, we relied on a single measurement of 25(OH)D and HOMA-IR and did not perform repeated assessments or use gold-standard methods for insulin sensitivity. Fifth, some metabolic variables, including 25(OH)D and HOMA-IR, demonstrated skewed distributions, and although efforts were made to apply appropriate statistical methods, our study may have been underpowered to detect small differences, and residual measurement and analytical error cannot be excluded. Finally, the lack of formal multivariable adjustment and the absence of detailed data on missing values limit internal validity and transparency, and should be considered when interpreting the findings.

## Conclusions

In this cohort of adults with T2DM from a tertiary care center in Pakistan, vitamin D deficiency was common and was associated with poorer glycemic profiles and higher estimated insulin resistance. However, the strength of these associations was modest, and the cross-sectional design does not allow causal inferences or evaluation of treatment effects. The findings support consideration of vitamin D status as one of several factors related to metabolic control in T2DM, but should not be interpreted as evidence that vitamin D supplementation alone will improve glycemic outcomes. Given the potential for confounding by BMI, lifestyle factors, medication use, and sunlight exposure, as well as the weak magnitude of the observed correlations, clinical recommendations regarding screening or supplementation cannot be based on this study alone. Future longitudinal and interventional studies in similar populations, incorporating careful adjustment for adiposity, lifestyle determinants, and other confounders, are needed to clarify the clinical relevance of vitamin D in the comprehensive management of T2DM.

## Appendices

Data Collection Proforma

Study Title: Association of Vitamin D Deficiency With Glycemic Control in Type 2 Diabetes Mellitus

Researcher: Anwar Ul Haq

Hospital: Hayatabad Medical Complex, Peshawar

Variable/Data

Patient ID ______

Age (years) ______

Gender (M/F) ______

Height (cm) ______

Weight (kg) ______

BMI (kg/m²) ______

Duration of Diabetes (years) ______

Current Diabetes Treatment (OHA/Insulin/Both) ______

Blood Pressure (mmHg) SBP: ____ / DBP: ____

Serum 25(OH) Vitamin D (ng/mL) ______

Vitamin D Status (Deficient / Sufficient) ______

HbA1c (%) ______

Fasting Plasma Glucose (mg/dL) ______

Postprandial Glucose (mg/dL) ______

Fasting Insulin (µU/mL) ______

HOMA-IR ______

Notes / Comments __________________________________
